# Supplementary material for: Associations between Google Search Trends for Symptoms and COVID-19 Confirmed and Death Cases in the United States
Source: Int J Environ Res Public Health. 2021 Apr 25;18(9):4560. doi: 10.3390/ijerph18094560 (PMC8123439; doi:10.3390/ijerph18094560)
Supplement: Supplementary file 1 [file ijerph-18-04560-s001.zip › Additional file 1.pdf]

Supplementary Tables

Table S1. Dynamic correlations between Google trends for symptoms and COVID-19 confirmed cases.

Symptom	Dynamic correlation
Hypoxemia	0.7824
Ageusia	0.6935
Anosmia	0.6934
Dysgeusia	0.6477
Hypoxia	0.6356
Sinusitis	0.6218
Fever	0.6148
Low.grade.fever	0.6066
Xeroderma	0.5588
Pneumonia	0.5508
Chills	0.5422
Shortness.of.breath	0.5419
Cough	0.5360
Nasal.congestion	0.5227
Common.cold	0.5014
Chest.pain	0.4921
Nosebleed	0.4916
Eye.pain	0.4887
Shivering	0.4673
Phlegm	0.4666
Wheeze	0.4419
Myalgia	0.4371
Bronchitis	0.4270
Cheilitis	0.4198
Asthma	0.4120
Myocardial.infarction	0.4120
Headache	0.4025
Pleurisy	0.4009
Rhinorrhea	0.3988
Tachycardia	0.3957
Hypercapnia	0.3801
Sore.throat	0.3795
Panic.attack	0.3768
Hypochondriasis	0.3590
Angina.pectoris	0.3523
Snoring	0.3513
Tinnitus	0.3472
Middle.back.pain	0.3442
Acute.bronchitis	0.3422
Post.nasal.drip	0.3415

Sputum	0.3382
Anxiety	0.3376
Sleep.deprivation	0.3332
Bradycardia	0.3319
Infection	0.3308
Dandruff	0.3266
Palpitations	0.3221
Periorbital.puffiness	0.3218
Migraine	0.3207
Coma	0.3158
Cluster.headache	0.3133
Hemoptysis	0.3118
Onychorrhexis	0.3000
Stroke	0.2966
Burning.mouth.syndrome	0.2908
Halitosis	0.2834
Indigestion	0.2812
Inflammation	0.2787
Tonsillitis	0.2767
Night.sweats	0.2736
Upper.respiratory.tract.infection	0.2721
Pulmonary.edema	0.2685
Unconsciousness	0.2643
Tremor	0.2640
Arthralgia	0.2634
Heartburn	0.2617
Trichoptilosis	0.2604
Flatulence	0.2601
Croup	0.2600
Insomnia	0.2577
Hyperventilation	0.2576
Sleep.disorder	0.2550
Nasal.polyp	0.2456
Rhinitis	0.2442
Hair.loss	0.2416
Hyperthermia	0.2391
Weakness	0.2333
Tenderness	0.2321
Pericarditis	0.2316
Impulsivity	0.2307
Beau.s.lines	0.2259
Blepharospasm	0.2259
hyperhidrosis	0.2247
Globus.pharyngis	0.2215

Generalized.anxiety.disorder	0.2183
Cyanosis	0.2170
Hypotension	0.2156
Abdominal.obesity	0.2121
Acne	0.2121
Binge.eating	0.2110
Cataplexy	0.2107
Shallow.breathing	0.2104
Throat.irritation	0.2071
Impetigo	0.2069
Red.eye	0.2056
Eye.strain	0.2050
Gastroesophageal.reflux.disease	0.2028
Asperger.syndrome	0.2000
Sleep.apnea	0.1977
Dry.eye.syndrome	0.1968
Major.depressive.disorder	0.1884
Rosacea	0.1855
Amnesia	0.1827
Sharp.pain	0.1822
Arthritis	0.1814
Tachypnea	0.1812
Aphasia	0.1794
Pleural.effusion	0.1785
Confusion	0.1776
Gingivitis	0.1774
Vomiting	0.1768
Hypersomnia	0.1765
Epiphora	0.1764
Hypoglycemia	0.1744
Fibromyalgia	0.1743
Convulsion	0.1742
Attention.deficit.hyperactivity.disorder	0.1716
Yawn	0.1713
Encephalopathy	0.1679
Diabetic.ketoacidosis	0.1656
Depression	0.1643
Blushing	0.1601
Hypertension	0.1587
Polydipsia	0.1537
Jaundice	0.1502
Ventricular.fibrillation	0.1481
Oral.candidiasis	0.1477
Visual.acuity	0.1435

Heart.arrhythmia	0.1427
Bleeding.on.probing	0.1422
Suicidal.ideation	0.1420
Shyness	0.1419
Depersonalization	0.1380
Xerostomia	0.1379
Male.infertility	0.1359
Stridor	0.1356
Grandiosity	0.1354
Burning.Chest.Pain	0.1351
Erythema	0.1346
Neonatal.jaundice	0.1331
Sensitivity.to.sound	0.1309
Mood.disorder	0.1286
Hyperglycemia	0.1281
Astigmatism	0.1278
Eczema	0.1269
Lightheadedness	0.1262
Cleft.lip.and.cleft.palate	0.1259
Splenomegaly	0.1255
Apnea	0.1247
Self.harm	0.1202
Stomach.rumble	0.1195
Uterine.contraction	0.1192
Crackles	0.1167
Bleeding	0.1156
Developmental.disability	0.1155
Ataxia	0.1140
Nausea	0.1135
Psychosis	0.1132
Diabetes	0.1127
Chancre	0.1089
Pyelonephritis	0.1071
Sexual.dysfunction	0.1061
Otitis.media	0.1055
Focal.seizure	0.1050
Congenital.heart.defect	0.1040
Avoidant.personality.disorder	0.1018
Hypertrophy	0.1015
Guilt	0.1000
Blurred.vision	0.0995
Leukorrhea	0.0985
Dysphagia	0.0980
Melena	0.0975

Food.craving	0.0969
Rectal.prolapse	0.0932
Generalized.tonicâ..clonic.seizure	0.0897
Insulin.resistance	0.0883
Compulsive.behavior	0.0876
Viral.pneumonia	0.0875
Hyperkalemia	0.0857
Osteoporosis	0.0838
Type.2.diabetes	0.0831
Excessive.daytime.sleepiness	0.0805
Strabismus	0.0795
Heart.murmur	0.0777
Dysautonomia	0.0775
Toothache	0.0742
Asphyxia	0.0723
Chorea	0.0722
Conjunctivitis	0.0717
Subdural.hematoma	0.0715
Anemia	0.0693
Kidney.failure	0.0674
Fatigue	0.0663
Rumination	0.0646
Hypokalemia	0.0643
Meningitis	0.0633
Testicular.pain	0.0626
Hiccup	0.0623
Anal.fissure	0.0613
Diarrhea	0.0611
Pulmonary.hypertension	0.0611
Hypomania	0.0566
Ear.pain	0.0562
Biliary.colic	0.0556
Muscle.atrophy	0.0549
Myoclonus	0.0538
Polycythemia	0.0506
Mouth.ulcer	0.0502
Thrombocytopenia	0.0492
Hematochezia	0.0489
Seborrheic.dermatitis	0.0486
Iron.deficiency	0.0476
Cardiac.arrest	0.0468
Crepitus	0.0466
Constipation	0.0464
Intracranial.pressure	0.0445

Chronic.pain	0.0430
Perspiration	0.0427
Morning.sickness	0.0400
Esophagitis	0.0390
Bacterial.vaginosis	0.0375
Hypocalcaemia	0.0366
Ptosis	0.0350
Angular.cheilitis	0.0336
Erectile.dysfunction	0.0328
Fasciculation	0.0297
Neck.mass	0.0280
pancreatitis	0.0264
Hypogonadism	0.0261
Auditory.hallucination	0.0254
Hyperpigmentation	0.0254
Epilepsy	0.0252
Hyperemesis.gravidarum	0.0250
Clouding.of.consciousness	0.0240
Orthostatic.hypotension	0.0221
Neck.pain	0.0206
Postural.orthostatic.tachycardia.syndrome	0.0197
Spasticity	0.0195
Hyponatremia	0.0188
Syncope	0.0163
Photophobia	0.0161
Urethritis	0.0161
Hemolysis	0.0135
Nystagmus	0.0108
Hay.fever	0.0107
Vaginal.discharge	0.0106
Ventricular.tachycardia	0.0083
Stuttering	0.0024
Paresthesia	-0.0021
Neuralgia	-0.0036
Abdominal.pain	-0.0057
Kyphosis	-0.0076
Gastroparesis	-0.0078
Aphonia	-0.0084
Laryngitis	-0.0103
Paranoia	-0.0133
Atheroma	-0.0142
Autoimmune.disease	-0.0142
Pain	-0.0147
Hepatotoxicity	-0.0148

Hypermobility	-0.0162
Canker.sore	-0.0173
Low.back.pain	-0.0177
Compulsive.hoarding	-0.0180
Hydrocephalus	-0.0180
Intermenstrual.bleeding	-0.0193
Blood.in.stool	-0.0203
Dystonia	-0.0210
Periodontal.disease	-0.0211
Kidney.stone	-0.0252
Night.terror	-0.0257
Amenorrhea	-0.0259
Otitis	-0.0268
Swollen.lymph.nodes	-0.0300
Braxton.Hicks.contractions	-0.0305
Dysmenorrhea	-0.0320
Skin.ulcer	-0.0323
Poor.posture	-0.0334
Hyperlipidemia	-0.0343
Vasculitis	-0.0351
Ascites	-0.0359
Rectal.pain	-0.0360
Fibrillation	-0.0408
Dizziness	-0.0411
Alcoholism	-0.0428
Dentin.hypersensitivity	-0.0438
Hot.flash	-0.0442
Hypertriglyceridemia	-0.0445
Urinary.urgency	-0.0456
Weight.gain	-0.0476
Lactose.intolerance	-0.0486
Encephalitis	-0.0503
Manic.Disorder	-0.0504
Gingival.recession	-0.0516
Tic	-0.0523
Fecal.incontinence	-0.0553
Irregular.menstruation	-0.0568
Hepatic.encephalopathy	-0.0576
Allergy	-0.0590
Scoliosis	-0.0665
Hives	-0.0711
Muscle.weakness	-0.0715
Dyspareunia	-0.0725
Amblyopia	-0.0741

Mitral.insufficiency	-0.0749
Delayed.onset.muscle.soreness	-0.0751
Hypercalcaemia	-0.0764
Nocturnal.enuresis	-0.0774
Folate.deficiency	-0.0775
Dementia	-0.0778
Hypothyroidism	-0.0778
Rheum	-0.0791
Mood.swing	-0.0795
Allergic.conjunctivitis	-0.0801
Bowel.obstruction	-0.0831
Facial.nerve.paralysis	-0.0850
Facial.swelling	-0.0880
Vertigo	-0.0892
Osteopenia	-0.0916
Round.ligament.pain	-0.0917
Food.intolerance	-0.0931
Bruxism	-0.0952
Neutropenia	-0.0958
Prediabetes	-0.0974
Photopsia	-0.0983
Desquamation	-0.0992
Angioedema	-0.1034
Implantation.bleeding	-0.1037
Goitre	-0.1049
Renal.colic	-0.1063
Pelvic.inflammatory.disease	-0.1074
Back.pain	-0.1075
Melasma	-0.1095
Otitis.externa	-0.1104
Vaginitis	-0.1109
Bell.s.palsy	-0.1136
Pus	-0.1146
Urinary.tract.infection	-0.1150
Bloating	-0.1157
Granuloma	-0.1198
Bone.tumor	-0.1203
Dermatitis	-0.1214
Urinary.incontinence	-0.1216
Mydriasis	-0.1297
Hepatitis	-0.1298
Malabsorption	-0.1298
Candidiasis	-0.1312
Photodermatitis	-0.1355

Anaphylaxis	-0.1372
Boil	-0.1410
Petechia	-0.1413
Adrenal.crisis	-0.1415
Hip.pain	-0.1415
Tumor	-0.1460
Dysphoria	-0.1472
Hemorrhoids	-0.1487
Fibrocystic.breast.changes	-0.1493
Hypercholesterolemia	-0.1506
Breast.pain	-0.1523
Polyuria	-0.1536
Vaginal.bleeding	-0.1540
Obesity	-0.1612
Cataract	-0.1613
Balance.disorder	-0.1622
Thyroid.nodule	-0.1630
Dysuria	-0.1640
Proteinuria	-0.1652
Underweight	-0.1681
Floater	-0.1712
Hematoma	-0.1738
Papule	-0.1750
Hematuria	-0.1753
Water.retention	-0.1864
Breakthrough.bleeding	-0.1884
Scar	-0.1899
Cirrhosis	-0.1912
Motion.sickness	-0.1963
Hyperthyroidism	-0.1983
Menorrhagia	-0.2033
Fatty.liver.disease	-0.2035
Pruritus.ani	-0.2154
Carpal.tunnel.syndrome	-0.2181
Polyneuropathy	-0.2187
Radiculopathy	-0.2220
Restless.legs.syndrome	-0.2322
Milium	-0.2340
Osteophyte	-0.2353
Colitis	-0.2469
Purpura	-0.2560
Itch	-0.2640
Cramp	-0.2654
Sciatica	-0.2663

Peripheral.neuropathy	-0.2664
Nerve.injury	-0.2680
Lymphedema	-0.2703
Skin.rash	-0.2734
Frequent.urination	-0.2779
Stretch.marks	-0.2786
Inflammatory.bowel.disease	-0.2811
Epidermoid.cyst	-0.2854
Nodule	-0.3028
Skin.condition	-0.3071
Leg.cramps	-0.3076
Knee.Pain	-0.3140
Bone.fracture	-0.3222
Erythema.chronicum.migrans	-0.3312
Ingrown.hair	-0.3364
Burn	-0.3375
Telangiectasia	-0.3746
Edema	-0.3756
Gout	-0.3760
Skin.tag	-0.3987
Lesion	-0.4102
Bunion	-0.4106
Podalgia	-0.4149
Swelling	-0.4292
Genital.wart	-0.4293
Actinic.keratosis	-0.4377
Swollen.feet	-0.4607
Varicose.veins	-0.4822
Wart	-0.4863
Bruise	-0.5156

Table S2. Dynamic correlations between Google trends for symptoms and COVID-19 death cases.

Symptom	Dynamic correlation
Hypoxemia	0.6470
Hypoxia	0.5358
Pneumonia	0.4816
Anosmia	0.4562
Ageusia	0.4120
Panic.attack	0.4024
Fever	0.4017
Shortness.of.breath	0.3986
Chest.pain	0.3974
Dysgeusia	0.3871
Bradycardia	0.3576
Bronchitis	0.3542
Chills	0.3510
Sinusitis	0.3505
Tachycardia	0.3502
Common.cold	0.3473
Xeroderma	0.3465
Cough	0.3460
Wheeze	0.3415
Low.grade.fever	0.3406
Abdominal.obesity	0.3377
Pleurisy	0.3314
Anxiety	0.3273
Dandruff	0.3260
Nasal.congestion	0.3130
Phlegm	0.3126
Beau.s.lines	0.3098
Shivering	0.3084
Nosebleed	0.2941
Onychorrhexis	0.2937
Periorbital.puffiness	0.2916
Myocardial.infarction	0.2889
Middle.back.pain	0.2832
Eye.pain	0.2823
Sleep.deprivation	0.2803
Impulsivity	0.2794
Trichoptilosis	0.2792
Tinnitus	0.2736
Asthma	0.2729
Acute.bronchitis	0.2712
Yawn	0.2617
Hyperventilation	0.2595

Binge.eating	0.2587
Palpitations	0.2545
Hair.loss	0.2522
Insomnia	0.2467
Hypercapnia	0.2466
Tremor	0.2457
Coma	0.2450
Pulmonary.edema	0.2429
Uterine.contraction	0.2413
Headache	0.2391
Hypotension	0.2360
Myalgia	0.2334
Sleep.disorder	0.2302
Angina.pectoris	0.2268
Heartburn	0.2226
Hyperthermia	0.2203
Tachypnea	0.2201
Sore.throat	0.2144
Unconsciousness	0.2143
Indigestion	0.2141
Generalized.anxiety.disorder	0.2119
Guilt	0.2092
Neonatal.jaundice	0.2060
Halitosis	0.2055
Cheilitis	0.2010
Self.harm	0.2010
Infection	0.1992
Night.sweats	0.1983
Confusion	0.1953
Hemoptysis	0.1950
Inflammation	0.1933
Eye.strain	0.1913
Tenderness	0.1898
Gastroesophageal.reflux.disease	0.1886
hyperhidrosis	0.1876
Snoring	0.1846
Sputum	0.1831
Erythema	0.1814
Shyness	0.1805
Globus.pharyngis	0.1787
Cluster.headache	0.1779
Hypochondriasis	0.1777
Stroke	0.1764
Attention.deficit.hyperactivity.disorder	0.1762

Hiccup	0.1752
Pericarditis	0.1735
Psychosis	0.1700
Tonsillitis	0.1697
Migraine	0.1687
Asperger.syndrome	0.1646
Shallow.breathing	0.1645
Depersonalization	0.1640
Grandiosity	0.1611
Compulsive.behavior	0.1606
Arthritis	0.1574
Burning.Chest.Pain	0.1544
Flatulence	0.1544
Sharp.pain	0.1522
Depression	0.1517
Auditory.hallucination	0.1512
Perspiration	0.1478
Crackles	0.1474
Suicidal.ideation	0.1467
Rhinorrhea	0.1458
Stridor	0.1430
Croup	0.1413
Muscle.atrophy	0.1399
Amnesia	0.1392
Sensitivity.to.sound	0.1384
Eczema	0.1376
Jaundice	0.1375
Acne	0.1365
Aphasia	0.1336
Post.nasal.drip	0.1313
Convulsion	0.1309
Major.depressive.disorder	0.1304
Burning.mouth.syndrome	0.1277
Rosacea	0.1265
Bleeding.on.probing	0.1260
Oral.candidiasis	0.1260
Hyperglycemia	0.1234
Hypoglycemia	0.1224
Upper.respiratory.tract.infection	0.1221
Blushing	0.1212
Gingivitis	0.1191
Visual.acuity	0.1181
Chorea	0.1164
Rhinitis	0.1163

Seborrheic.dermatitis	0.1147
Cyanosis	0.1123
Viral.pneumonia	0.1122
Insulin.resistance	0.1109
Hyperpigmentation	0.1087
Encephalopathy	0.1068
Weakness	0.1068
Lightheadedness	0.1065
Poor.posture	0.1051
Sleep.apnea	0.1029
Crepitus	0.1008
Syncope	0.1006
Rumination	0.0973
Neck.mass	0.0938
Heart.arrhythmia	0.0931
Braxton.Hicks.contractions	0.0924
Diabetes	0.0924
Stomach.rumble	0.0924
Mood.disorder	0.0906
Ventricular.fibrillation	0.0900
Impetigo	0.0892
Anal.fissure	0.0864
Hypertension	0.0864
Otitis.media	0.0852
Pleural.effusion	0.0827
Cleft.lip.and.cleft.palate	0.0822
Avoidant.personality.disorder	0.0793
Vomiting	0.0793
Nasal.polyp	0.0789
Tic	0.0784
Hypokalemia	0.0758
Biliary.colic	0.0741
Congenital.heart.defect	0.0739
Focal.seizure	0.0734
Throat.irritation	0.0731
Orthostatic.hypotension	0.0710
Apnea	0.0688
Red.eye	0.0678
Splenomegaly	0.0661
Hyperkalemia	0.0646
Diabetic.ketoacidosis	0.0636
Diarrhea	0.0622
Ataxia	0.0619
Polydipsia	0.0583

Xerostomia	0.0551
Kyphosis	0.0545
Epiphora	0.0520
Intracranial.pressure	0.0520
Compulsive.hoarding	0.0505
Kidney.failure	0.0496
Developmental.disability	0.0483
Conjunctivitis	0.0475
Hypersomnia	0.0454
Constipation	0.0441
Testicular.pain	0.0425
Blepharospasm	0.0417
Weight.gain	0.0406
Vasculitis	0.0401
Cataplexy	0.0399
Arthralgia	0.0377
Ear.pain	0.0362
Nausea	0.0339
Astigmatism	0.0335
Rectal.pain	0.0313
Hay.fever	0.0304
Angular.cheilitis	0.0289
Hematochezia	0.0288
Myoclonus	0.0276
Asphyxia	0.0270
Spasticity	0.0265
Blurred.vision	0.0261
Cardiac.arrest	0.0259
Paranoia	0.0259
Photophobia	0.0237
Generalized.tonicâ..clonic.seizure	0.0234
Dry.eye.syndrome	0.0233
Male.infertility	0.0232
Ventricular.tachycardia	0.0230
Polycythemia	0.0226
Clouding.of.consciousness	0.0196
Melena	0.0193
Anemia	0.0171
Rectal.prolapse	0.0167
Hemolysis	0.0165
Excessive.daytime.sleepiness	0.0143
Swollen.lymph.nodes	0.0104
Blood.in.stool	0.0073
Subdural.hematoma	0.0067

Hypermobility	0.0060
Neuralgia	0.0049
Hypomania	0.0039
Heart.murmur	0.0038
Low.back.pain	0.0034
Esophagitis	0.0017
Type.2.diabetes	0.0014
Melasma	0.0010
Amenorrhea	-0.0003
Night.terror	-0.0008
Allergic.conjunctivitis	-0.0037
Nystagmus	-0.0039
Fasciculation	-0.0043
Fatigue	-0.0050
Osteoporosis	-0.0055
Aphonia	-0.0058
Fibromyalgia	-0.0066
Abdominal.pain	-0.0072
Postural.orthostatic.tachycardia.syndrome	-0.0077
Thrombocytopenia	-0.0078
Pain	-0.0081
Epilepsy	-0.0082
Gastroparesis	-0.0087
Toothache	-0.0090
Dysautonomia	-0.0097
Ptosis	-0.0099
Hypocalcaemia	-0.0101
Rheum	-0.0120
Bleeding	-0.0123
Otitis	-0.0133
Paresthesia	-0.0133
Chronic.pain	-0.0168
Atheroma	-0.0182
Laryngitis	-0.0185
Meningitis	-0.0187
Hepatic.encephalopathy	-0.0222
Gingival.recession	-0.0259
Hyponatremia	-0.0264
Mouth.ulcer	-0.0267
Water.retention	-0.0272
Dysphoria	-0.0273
Dizziness	-0.0277
Dysphagia	-0.0284
Leukorrhea	-0.0308

Manic.Disorder	-0.0322
Delayed.onset.muscle.soreness	-0.0325
Iron.deficiency	-0.0326
Neck.pain	-0.0327
Mood.swing	-0.0332
Hypertrophy	-0.0347
Food.craving	-0.0348
Chancre	-0.0358
Lactose.intolerance	-0.0366
Stuttering	-0.0384
Papule	-0.0413
Strabismus	-0.0431
Hyperemesis.gravidarum	-0.0449
Photodermatitis	-0.0449
Hydrocephalus	-0.0453
Pyelonephritis	-0.0461
Autoimmune.disease	-0.0471
Dystonia	-0.0492
Facial.swelling	-0.0492
Adrenal.crisis	-0.0497
Hives	-0.0503
Bloating	-0.0508
Allergy	-0.0523
Breast.pain	-0.0566
Hypertriglyceridemia	-0.0574
Sexual.dysfunction	-0.0608
Dermatitis	-0.0627
Facial.nerve.paralysis	-0.0643
Pulmonary.hypertension	-0.0653
Folate.deficiency	-0.0658
Round.ligament.pain	-0.0667
Dyspareunia	-0.0694
Muscle.weakness	-0.0716
Skin.ulcer	-0.0718
Petechia	-0.0730
Kidney.stone	-0.0761
Canker.sore	-0.0768
Food.intolerance	-0.0780
Vertigo	-0.0786
Pus	-0.0795
Dysmenorrhea	-0.0801
Amblyopia	-0.0805
Desquamation	-0.0805
Underweight	-0.0887

Ascites	-0.0897
Nocturnal.enuresis	-0.0939
Hepatotoxicity	-0.0953
Polyuria	-0.0958
Angioedema	-0.0965
pancreatitis	-0.0993
Hyperlipidemia	-0.0994
Encephalitis	-0.0996
Scar	-0.1017
Goitre	-0.1022
Back.pain	-0.1035
Dementia	-0.1066
Otitis.externa	-0.1070
Prediabetes	-0.1074
Mitral.insufficiency	-0.1076
Stretch.marks	-0.1083
Morning.sickness	-0.1100
Fibrocystic.breast.changes	-0.1110
Hemorrhoids	-0.1112
Obesity	-0.1135
Dentin.hypersensitivity	-0.1136
Bell.s.palsy	-0.1164
Renal.colic	-0.1166
Hot.flash	-0.1176
Bowel.obstruction	-0.1199
Mydriasis	-0.1212
Scoliosis	-0.1226
Irregular.menstruation	-0.1244
Hypothyroidism	-0.1252
Hypercalcaemia	-0.1257
Restless.legs.syndrome	-0.1270
Hip.pain	-0.1310
Purpura	-0.1317
Erectile.dysfunction	-0.1340
Urinary.urgency	-0.1355
Alcoholism	-0.1382
Fecal.incontinence	-0.1383
Intermenstrual.bleeding	-0.1393
Urinary.incontinence	-0.1394
Malabsorption	-0.1418
Bruxism	-0.1448
Milium	-0.1464
Periodontal.disease	-0.1474
Boil	-0.1487

Hypogonadism	-0.1489
Fibrillation	-0.1491
Balance.disorder	-0.1494
Osteopenia	-0.1541
Floater	-0.1563
Granuloma	-0.1576
Edema	-0.1604
Burn	-0.1648
Skin.condition	-0.1658
Tumor	-0.1659
Vaginal.discharge	-0.1698
Knee.Pain	-0.1743
Carpal.tunnel.syndrome	-0.1756
Photopsia	-0.1765
Urethritis	-0.1772
Telangiectasia	-0.1778
Anaphylaxis	-0.1779
Polyneuropathy	-0.1790
Skin.rash	-0.1835
Neutropenia	-0.1854
Candidiasis	-0.1884
Cramp	-0.1892
Bone.tumor	-0.1897
Erythema.chronicum.migrans	-0.1919
Hyperthyroidism	-0.1952
Dysuria	-0.1957
Cataract	-0.1973
Hematoma	-0.1981
Cirrhosis	-0.2031
Hepatitis	-0.2037
Implantation.bleeding	-0.2050
Sciatica	-0.2075
Bone.fracture	-0.2084
Itch	-0.2104
Bacterial.vaginosis	-0.2124
Motion.sickness	-0.2133
Swollen.feet	-0.2145
Pruritus.ani	-0.2146
Colitis	-0.2163
Proteinuria	-0.2211
Urinary.tract.infection	-0.2236
Peripheral.neuropathy	-0.2257
Hypercholesterolemia	-0.2272
Osteophyte	-0.2294

Pelvic.inflammatory.disease	-0.2308
Epidermoid.cyst	-0.2317
Frequent.urination	-0.2330
Ingrown.hair	-0.2384
Menorrhagia	-0.2401
Varicose.veins	-0.2404
Fatty.liver.disease	-0.2414
Skin.tag	-0.2437
Thyroid.nodule	-0.2471
Swelling	-0.2518
Nerve.injury	-0.2540
Vaginal.bleeding	-0.2590
Inflammatory.bowel.disease	-0.2629
Lymphedema	-0.2632
Podalgia	-0.2659
Gout	-0.2691
Leg.cramps	-0.2732
Actinic.keratosis	-0.2853
Vaginitis	-0.2899
Radiculopathy	-0.2949
Breakthrough.bleeding	-0.3050
Bunion	-0.3120
Lesion	-0.3160
Hematuria	-0.3209
Wart	-0.3227
Bruise	-0.3308
Genital.wart	-0.3731
Nodule	-0.3829
